# Subtilisin-like protease-1 secreted through type IV secretion system contributes to high virulence of *Streptococcus suis* 2

**DOI:** 10.1038/srep27369

**Published:** 2016-06-08

**Authors:** Supeng Yin, Ming Li, Xiancai Rao, Xinyue Yao, Qiu Zhong, Min Wang, Jing Wang, Yizhi Peng, Jiaqi Tang, Fuquan Hu, Yan Zhao

**Affiliations:** 1 Department of Microbiology, Third Military Medical University, Chongqing, China; 2 Institute of Burn Research, Southwest Hospital, Third Military Medical University, Chongqing, China; 3 PLA Research Institute of Clinical Laboratory Medicine, Nanjing general hospital of Nanjing Military command, Nanjing 210002, China

## Abstract

*Streptococcus suis* serotype 2 is an emerging zoonotic pathogen that triggered two outbreaks of streptococcal toxic shock syndrome (STSS) in China. Our previous research demonstrated that a type IV secretion system (T4SS) harbored in the 89K pathogenicity island contributes to the pathogenicity of *S. suis* 2. In the present study, a shotgun proteomics approach was employed to identify the effectors secreted by T4SS in *S. suis* 2, and surface-associated subtilisin-like protease-1 (SspA-1) was identified as a potential virulence effector. Western blot analysis and pull-down assay revealed that SspA-1 secretion depends on T4SS. Knockout mutations affecting *sspA-1* attenuated *S. suis* 2 and impaired the pathogen’s ability to trigger inflammatory response in mice. And purified SspA-1 induced the secretion of IL-6, TNF-α, and IL-12p70 in THP-1 cells directly. SspA-1 is the first T4SS virulence effector reported in Gram-positive bacteria. Overall, these findings allow us to gain further insights into the pathogenesis of T4SS and STSS.

Bacteria are equipped with various mechanisms to secrete proteins essential for their pathogenicity and survival. In addition to the typical general secretion (Sec) and twin-arginine translocation (Tat) systems^1^, at least seven specialized protein-secretion systems have been reported in bacteria^2^,^3^,^4^. Among the known secretion systems, type IV secretion systems (T4SSs) are the most versatile and widespread in archaea and bacteria^5^. T4SSs are also unique because of their ability to transport DNA substrates, and pathogenic effectors across the cell envelope. However, studies on T4SSs have mainly focused on Gram-negative bacteria, such as the VirB/D4 system from *Agrobacterium tumefaciens* and closely related systems from *Escherichia coli* encoded by conjugative plasmids^6^. Hence, limited information is available regarding Gram-positive T4SSs and their substrates.

*Streptococcus suis* serotype 2 (*S. suis* 2) is a Gram-positive zoonotic pathogen responsible for a variety of life-threatening infections in humans and pigs, such as meningitis, pneumonia, arthritis, and septicaemia^7^,^8^. In the two human outbreaks caused by *S. suis* 2 in China (1998 and 2005), a high proportion of patients manifested the typical symptoms of streptococcal toxic shock syndrome (STSS) characterized by a very short disease course and high mortality^9^,^10^. As such, the emergence of highly pathogenic *S. suis* 2 poses a serious threat to public health.

However, the pathogenetic mechanisms employed by the highly pathogenic *S. suis* 2 have yet to be clarified. Chen *et al.*^11^ conducted a comparative genomics research and found that a unique pathogenicity island (PAI) designated as 89K is specific to the two epidemic strains, namely, 98HAH12 and 05ZYH33, which caused the STSS outbreaks. We demonstrated that a functional VirB/D4 T4SS located at the 5′-end of 89 K mediates the conjugal transfer of 89 K, and this T4SS may secrete some unknown effectors triggering an excessive host inflammatory response and inducing STSS^12^,^13^. Therefore, the repertoire of T4SS effectors should be identified to elucidate the pathogenic mechanisms of STSS caused by *S. suis* 2.

In the current study, a shotgun proteomics strategy^14^,^15^ was applied to analyze the secretome of *S. sui*s 2 and a T4SS-deficient mutant. A T4SS effector termed surface-associated subtilisin-like protease-1 (SspA-1), which belongs to the subtilase family, was identified using this strategy. SspA-1 can trigger an excessive inflammatory response in a mouse infection model and further promote STSS development. To our knowledge, SspA-1 is the first T4SS effector identified in Gram-positive bacteria. Overall, our research provided significant insights into the pathogenesis of the highly virulent *S. suis* 2 strain.

## Materials and Methods

### Bacterial strains, plasmids, and culture conditions

The bacterial strains and plasmids used in this study are listed in Table S1. *S. suis* 2 strains were cultured in Todd–Hewitt broth containing 2% yeast extract. *E. coli* strains were grown in Luria-Bertani medium. If necessary, antibiotics were added to the media with the following concentrations: 100 mg/L spectinomycin, 100 mg/L ampicillin, 50 mg/L kanamycin, 1 mg/L erythromycin for *S. suis*, and 250 mg/L erythromycin for *E. coli*.

### Preparation of culture supernatants and whole-cell proteins

Cultures of different *S. suis* 2 strains harvested in the late exponential growth phase were centrifuged at 10,000 × *g* for 10 min at 4 °C. Supernatants and cell pellets were prepared as follows. The supernatants were precipitated with acetone–trichloroacetic acid in accordance with previously described methods^16^. The cell pellets were washed with PBS, resuspended in a lysis buffer (50 mM Tris–HCl, 2 mM EDTA, 100 mM NaCl, 0.5% Triton X-100, 10 mg/ml lysozyme, and protease inhibitor cocktail at pH 8.5–9.0), and incubated at 37 °C for 4 h. After disruption was performed with three cycles of alternating ultrasound and freezing/thawing, the lysates were centrifuged at 2,000 × *g* for 5 min to remove debris. The resulting supernatants were collected as whole-cell proteins.

### LC-MS/MS analysis

The precipitated proteins from the culture supernatants of wild-type *S. suis* 2 05ZYH33 and T4SS-deficient mutant strain (Δ*virD4-89K*) were analyzed through LC-MS/MS to identify the putative proteins secreted via T4SS. Protein samples were separated through sodium dodecyl sulfate–polyacrylamide gel electrophoresis (SDS–PAGE), pretreated with trypsin, and analyzed through LC-MS/MS by using UltiMate3000 RSLCnano liquid chromatography/Bruker maxis 4G Q-TOF. The resulting peptide mass fingerprints were compared against the ORFs of the genome of 05ZYH33 by using Mascot and Mascot Daemon software (Matrix Science); matches with *P* < 0.05 were considered with high confidence. Signal peptides were predicted using SignalP 4.1 server.

### Construction of *sspA-1* knockout mutant and complemented CΔ*sspA-1* strain

The *sspA-1* mutant was generated through allelic replacement with a spectinomycin (*spc*) resistance gene cassette as previously described^17^. The *sspA-1* upstream flanking sequence (left arm) was cloned as an *Eco*R I/*Bam*H I fragment and the downstream flanking sequence (right arm) as a *Pst* I/*Hin*d III fragment at both sides of the spectinomycin resistance gene in the pUC18 plasmid. The primers used to amplify the left and right arms are listed in Table S2. The recombinant plasmid was electrotransformed into competent cells of 05ZYH33. Positive transformants, which were resistant to spectinomycin, were further confirmed through PCR and DNA sequencing. A double-crossover mutant of *sspA-1* was isolated and designated as Δ*sspA-1*.

A 5049 bp fragment containing *sspA-1* and the promotor sequence was divided into two sequential fragments and amplified from the 05ZYH33 chromosome by using the PCR primers C*sspA-1*-F/C*sspA-1*-MR and C*sspA-1*-MF/C*sspA-1*-R (Table S2). The resulting PCR products were digested with *Bam*H I/*Sph* I and *Sph* I/*Eco*R I and then cloned into *E. coli–S. suis* pVA838 shuttle vector successively, as a result, pVA838-*sspA-1* was generated. After the result was verified through DNA sequencing, the resulting plasmid was electrotransformed into Δ*sspA-1* and designated as the complemented strain CΔ*sspA-1*.

### Quantitative real-time PCR (qRT-PCR)

Total RNA of various *S. suis* 2 strains was extracted from cultures grown to the late exponential phase by using an SV total RNA isolation system (Promega). RNA was reverse transcribed to cDNA by using a Transcriptor first-strand cDNA synthesis kit (Roche). qRT-PCR was conducted using SYBR premix Ex TaqTM (TaKaRa) in an Eco Real-Time PCR System (Illumina). Levels of 16S rRNA were used as internal control^18^. The primers used for qRT-PCR are shown in Table S2. The fold changes of the *sspA-1* transcripts were quantified using a comparative threshold cycle (ΔΔC_T_) program in the Eco software package. The assays were performed in triplicate.

### Cloning, overexpression, and purification of recombinant proteins

The possible B-cell epitopes of SspA-1 were analyzed and a 2349 bp DNA fragment encoding all the predicted functional domains of SspA-1 was selected and cloned into the pET-28a expression vector by using the primers listed in Table S2. *E. coli* BL21 harboring the SspA-1-expressing plasmid was induced with 1 mM IPTG at 30 °C for 6 h. Cells were harvested and resuspended in PBS containing 1 mM PMSF. After disruption was performed through ultrasound in an ice bath, cell lysates were centrifuged, and supernatant was collected and filtered through a 0.45 μm membrane. His–SspA-1 in the cleared supernatant was purified using a His GraviTrap column (Bio-Rad) in accordance with the manufacturer’s instructions.

VirD4–89 K was overexpressed with the GST fusion vector pGEX-6P-1 in *E. coli* BL21, and this process was similar to that applied to induce His–SspA-1. GST–VirD4 and GST alone were purified using gravity columns (Bio-Rad) with a Uniflow glutathione resin in accordance with the manufacturer’s recommended protocol.

### Preparation of polyclonal antibodies against SspA-1

Five six-week-old BALB/c mice were immunized subcutaneously and intraperitoneally on days 0, 14, and 28 with 40 μg of recombinant SspA-1 formulated with a complete Freund adjuvant (Sigma) for the first dose, incomplete Freund adjuvant (Sigma) for the second dose, and SspA-1 alone for the third inoculation per mouse. Sera were collected from the mice at 7 days after the last immunization^19^. Western blot analysis and enzyme-linked immunosorbent assay (ELISA) were performed to determine the specificity and titers of the antibodies against SspA-1. The polyclonal antibodies against SspA-2 were prepared in the same manner. The animal experiments were performed in accordance with the International Guiding Principles for Biomedical Research involving Animals-1985 and approved by the Laboratory Animal Welfare and Ethics Committee of the Third Military Medical University.

### Western blot analysis

The protein concentrations in whole-cell lysates and culture supernatants were calculated using a Bradford protein assay kit (Beyotime) or a Bio-Rad DC protein assay kit. An equal amount of total protein from each sample was loaded and separated in 10% SDS–PAGE. The proteins were transferred to a polyvinylidene fluoride membrane through electrophoresis. The membrane was blocked in 5% milk for 1 h at room temperature and incubated with primary antibodies at 4 °C overnight. After the membrane was washed thrice in PBST buffer, the membrane was incubated with a horseradish-peroxidase-conjugated secondary antibody (ZSGB-BIO) for 1 h at room temperature. The signals were detected using SuperSignal West Pico (Pierce) and Image Station 4000MM PRO Digital Imaging System (Kodak). The primary antibodies used in this study were polyclonal mouse anti-SspA-1 (prepared in our laboratory), monoclonal mouse anti-RNA polymerase subunit RpoB (Abcam), and monoclonal mouse anti-GST antibody (ZSGB-BIO).

### GST pull-down assay

The purified GST–VirD4 or GST (negative control) was mixed with glutathione beads equilibrated with TBST (20 mM Tris–HCl, 200 mM NaCl, and 1% Tween 20 at pH 8.0) at 4 °C overnight. After the beads were washed and resuspended in TBST, the purified His–SspA-1 or total protein from Δ*virD4–89K* lysates, which contained the abundant native SspA-1, was added. Bovine serum albumin (BSA) and Δ*sspA-1* lysates were used as negative controls of the prey. The mixture was incubated with rotation at 4 °C overnight; the beads were then washed thoroughly with TBST. The final samples and the input samples were subjected to SDS–PAGE and Western blot to detect His–SspA-1, native SspA-1, GST, and GST–VirD4^20^,^21^.

### Virulence studies

A total of 40 four-week-old female BALB/c mice were randomly allocated to four groups (10 animals per group), and each mouse was challenged by intraperitoneally injecting wild-type *S. suis* 2, T4SS-deficient mutant (Δ*virD4-89K*), Δ*sspA-1*, or CΔ*sspA-1* strains at a dose of 2.5 × 10^7^ CFU/mice. Clinical signs and survival times were observed and recorded for 7 days after infection.

### Measurement of inflammatory cytokines in infected mice

A total of 60 four-week-old BALB/c mice were randomly assigned to three groups, and each mouse was injected intraperitoneally with wild-type *S. suis* 2, T4SS-deficient mutant (Δ*virD4-89K*), or Δ*sspA-1* at a dose of 10^6^ CFU/mouse. At 4, 8, 12, and 16 h after infection, five mice per group were euthanized, and serum samples were collected in accordance with previously described methods^13^. Cytokines were quantified using a Quantikine ELISA kit (R&D Systems) in accordance with the manufacturer’s recommendations. In addition, the bacterial loads in the blood samples of infected mice were determined by the drop plate method.

### Induction of inflammatory cytokines in THP-1 cells

The human leukemia monocytic cell line THP-1 was cultivated in RPMI-1640 medium supplemented with 10% heat-inactivated fetal bovine serum at 37°C in a 5% CO_2_ atmosphere and induced by PMA (200 ng/ml). When appropriate, cells were seeded in a 24-well plate (5 × 10^6^ cells/well) and treated separately with purified SspA-1 (100 μg/ml), Trypsin (100 μg/ml), SspA-1 (100 μg/ml) with polymyxin B (1 μg/ml), LPS (5 μg/ml), and PBS for 24 hours. Trypsin and LPS were negative control and positive control respectively. Polymyxin B was used to block the effect of any contaminating LPS in the purified SspA-1. After these treatments, the cell culture supernatants were collected and the cytokines were quantified as previously described.

### Immunization and challenge of mice

A total of 20 six-week-old female BALB/c mice were randomly assigned to two groups. Group 1 was immunized as described in the preparation of polyclonal antibodies against SspA-1. Group 2 was immunized in the same way except that the recombinant SspA-1 was replaced with PBS. Blood samples were collected before each vaccination to determine the antibody response. Mouse serum titers were determined through ELISA in accordance with previously described methods^22^. One week after the third vaccination, the mice were challenged intraperitoneally with wild-type *S. suis* 2 at a dose of 10^8^ CFU/mouse. Survival times were recorded for 7 days after infection.

### Statistical analysis

Data were analyzed using Wilcoxon rank sum test, Kruskal–Wallis test, or Nemenyi test as appropriate. A value of *P* < 0.05 was considered significant.

## Results

### Screening of putative effectors of T4SS

A shotgun proteomics strategy was applied to identify the T4SS-dependent effectors secreted into the extracellular matrix and compare the secretomes of wild-type *S. suis* 2 (05ZYH33) and a T4SS-deficient mutant (Δ*virD4-89K*), which was attenuated in virulence and defective in T4SS-dependent horizontal gene transfer^12^,^13^. The extracellular proteins from the wild-type and T4SS-deficient mutant cultures in the late exponential phase were identified through LC-MS/MS. Three independent experiments were performed. Only the proteins detected at least twice in the wild-type supernatant and absent in the T4SS-deficient mutant supernatants were considered as putative T4SS effectors. Seven proteins satisfied these criteria (Table 1). The six other candidates, except prolyl–tRNA synthetase, exhibited putative signal peptides; these candidates are also predicted as extracellularly secreted. Among these proteins, two subtilisin-like serine proteases designated as SspA-1 and SspA-2 which were both detected three times in the wild-type supernatants were examined. Some members of the subtilase family are virulence determinants of *S. suis* 2 and can trigger a proinflammatory response in macrophages^23^,^24^,^25^,^26^. Therefore, these two putative effectors may be correlated with the development of STSS caused by *S. suis* 2. As such, the two putative effectors were further examined.

### SspA-1 is secreted in a T4SS-dependent manner

**Figure 1:**
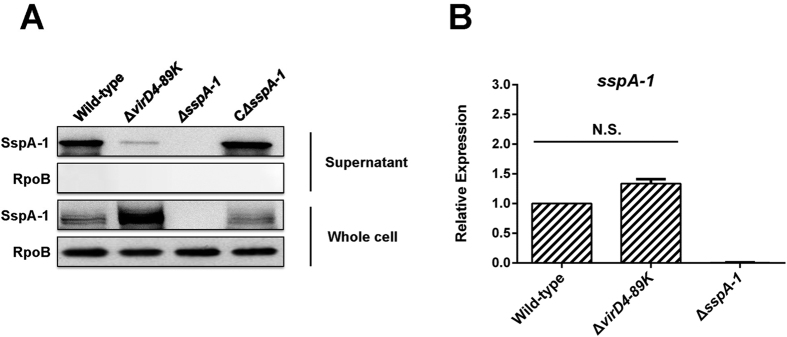
SspA-1 is secreted in a T4SS-dependent manner. (**A**) Western blot analysis comparing the levels of SspA-1 in the supernatants and cell pellets of the designated *S. suis* 2 strains. RNA polymerase subunit RpoB was used as loading control. (**B**) *sspA-1* transcripts quantified through real-time PCR. N. S. indicates no significant difference (*P *> 0.05) between *S. suis* 2 wild-type stain 05ZYH33 and T4SS-deficient mutant (Δ*virD4***–***89K*).

The culture supernatants and pellets from a wild-type strain, T4SS-deficient mutant (Δ*virD4-89K*), Δ*sspA-1*, or CΔ*sspA-1* were harvested and analyzed through Western blot with polyclonal antibodies against the two putative protein effectors. SspA-1 is abundant in the culture supernatants from the wild-type strain but is barely detected in those of Δ*virD4–89K* (Fig. 1A). By contrast, the reactivity bands of SspA-1 were significantly stronger in the cell pellets of Δ*virD4–89K* than in the wild-type strain. The results indicate that the deficiency of T4SS blocks the secretion of SspA-1 and leads to the accumulation of SspA-1 in the mutant cells. However, Western blot analysis revealed that the SspA-2 levels did not show any significant difference between the culture supernatants and pellets from *S. suis* 2 wild-type strain and T4SS-deficient mutant (data not shown). Thus, we focused on SspA-1.

RT-PCR assay was conducted to evaluate *sspA-1* transcription, as well as to exclude the possibility that T4SS may affect this transcription. As shown in Fig. 1B, the *sspA-1* transcription in the wild-type strain did not significantly differ from that of the T4SS-deficient mutant; therefore, various SspA-1 levels in the supernatants are not attributed to transcriptional differences.

### Interaction between SspA-1 and VirD4*–*89K

**Figure 2:**
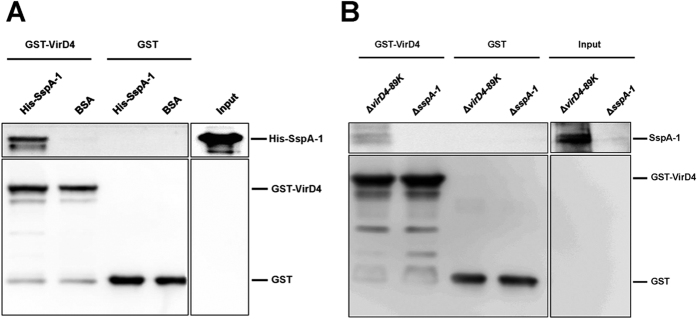
Direct interaction between SspA-1 and VirD4–89K. (**A**) Pull-down assay between GST**–**VirD4 and purified His**–**SspA-1. GST**–**VirD4 or GST alone was pre-absorbed with glutathione beads and mixed with purified His-SspA-1 or BSA. GST or BSA was used as a negative control for bait or prey, respectively. (**B**) Pull-down assay between GST**–**VirD4 and native SspA-1 in the Δ*virD4***–***89K* mutant. GST**–**VirD4 or GST alone was pre-absorbed with glutathione beads and then mixed with the total proteins from Δ*virD4***–***89K* or Δ*sspA-1* lysates. GST or Δ*sspA-1* lysates were used as negative controls for the bait or prey, respectively. Pull-down samples and input samples (0.1% input His-SspA-1 or 2% input Δ*virD4***–***89K* lysates) were subjected to Western blot with anti-SspA-1 or anti-GST.

In T4SS, VirD4 acts as a type 4 coupling protein, which binds T4SS substrates directly before these substrates are delivered into the protein channel. As a result, the interaction between SspA-1 and VirD4*–*89K was investigated through pull-down assays. In one assay, GST*–*VirD4 was incubated with a purified truncated poly-histidine-tagged SspA-1 (His*–*SspA-1), which contained all the predicted functional domains of SspA-1. In the other assay, native SspA-1, which exists in Δ*virD4–89K* lysates, was used instead of His–SspA-1. GST–VirD4 was co-precipitated with purified (Fig. 2A) or native (Fig. 2B) SspA-1; by contrast, GST alone as a negative control was not co-precipitated with either of the SspA-1 types. This finding suggested that SspA-1 directly interacts with VirD4–89K.

### Role of SspA-1 in the virulence of *S. suis*2

**Figure 3:**
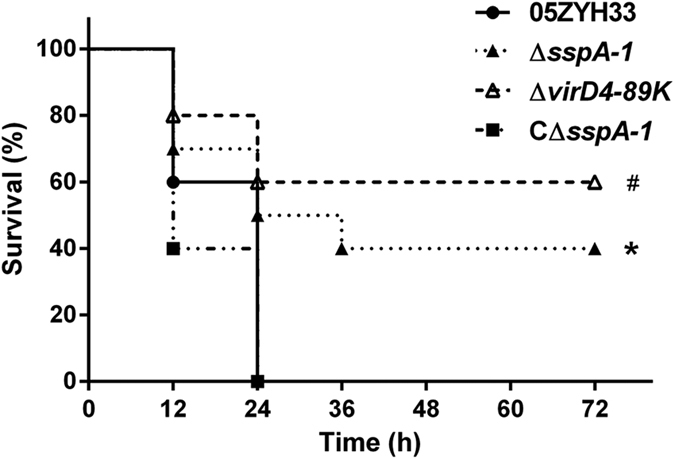
Survival curves of mice infected with the designated *S. suis* 2 strains. A total of 40 four-week-old female BALB/c mice were randomly allocated to four groups (10 animals per group) and challenged by intraperitoneally injecting a dose of 2.5 × 10^7^ CFU/mice. The results are representative of three independent experiments. *P < 0.05 for comparison between the mutant Δ*sspA-1* and the wild-type strain 05ZYH33 or CΔ*sspA-1* strain; **^#^***P* < 0.05 for comparison between the mutant Δ*virD4-89K* and the wild-type strain or CΔ*sspA-1*.

A *sspA-1* knockout mutant designated as Δ*sspA-1* and a complementary strain termed CΔ*sspA-1* were constructed to examine the role of SspA-1 in the pathogenicity of the highly virulent *S. suis* 2. The growth rate, chain length, capsular material thickness, and hemolytic activity of Δ*sspA-1* and CΔ*sspA-1* did not significantly differ from those of the wild-type strain (data not shown). BALB/c mice were used to assess the virulence of wild-type strain 05ZYH33, T4SS-deficient mutant (Δ*virD4-89K*), Δ*sspA-1*, and CΔ*sspA-1*. The mice inoculated with the wild-type strain manifested typical symptoms, such as rough hair coat, hypnesthesia, swollen eyes, and suppuration in the inner canthus but died within 24 h. By contrast, the mice infected with Δ*sspA-1* or Δ*virD4-89K* exhibited less serious symptoms and yielded 40% and 60% survival rates at the end of the experiment, respectively. When the mice were challenged with the complementary CΔ*sspA-1* strain, the results were similar to those obtained from the wild-type strain (Fig. 3). These results implied that SspA-1 contributes to the virulence of *S. suis* 2.

### Proinflammatory effect of SspA-1 *in vivo* and *in vitro*

**Figure 4:**
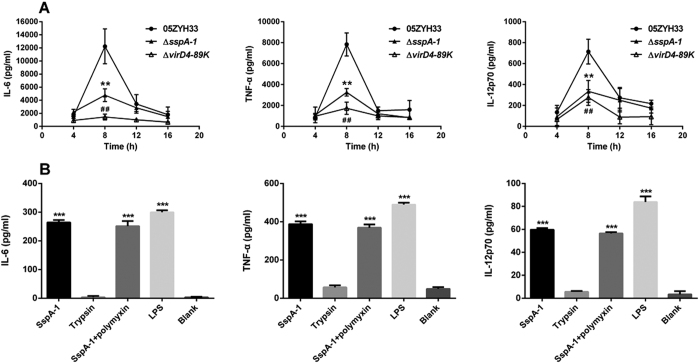
Cytokine levels in infected mice serum and culture supernatants of stimulated THP-1 cells. (**A**) Cytokine production in the BALB/c mice infected with the designated strains at 4, 8, 12, and 16 h after infection. **P < 0.01 for the comparison between the wild-type strain 05ZYH33 and Δ*sspA-1* mutant; ^##^*P* < 0.01 for the comparison between the wild-type strain 05ZYH33 and Δ*virD4–89K* mutant. (**B**) Cytokine production by stimulated THP-1 cells. ***P < 0.001 indicates a significant difference in comparison with the non-stimulated cells (Blank)c. All these data were obtained from three independent experiments.

We assessed the kinetic profiles of cytokine secretion in the mice infected with the wild-type strain, T4SS-deficient mutant (Δ*virD4*–*89K*), and Δ*sspA-1* at 4, 8, 12, and 16 h after infection. The serum levels of interleukin 6 (IL-6), tumor necrosis factor-α (TNF-α), and interleukin 12p70 (IL-12p70) from the three mouse groups showed a sharp peak at 8 h after infection (Fig. 4A); the peak values returned to basal levels at 12 or 16 h after infection. However, the IL-6, TNF-α, and IL-12p70 levels induced by either Δ*sspA-1* or Δ*virD4–89K* were markedly lower than those induced by the wild-type strain, particularly at 8 h after infection. The production of IL-6, TNF-α, and IL-12p70 in the mice infected with Δ*sspA-1* was slightly higher than that in mice infected with Δ*virD4*–*89K*; nevertheless, the two groups did not significantly differ from each other. Meanwhile, the bacterial loads in the blood samples of the three groups showed no significant difference at any of the same time point (Fig. S1).

*In vitro*, the treatment of purified recombinant SspA-1 resulted in a significant increase of IL-6, TNF-α, and IL-12p70 secretion in THP-1 cells while trypsin did not (Fig. 4B). And the proinflammatory effect of SspA-1 could not be blocked by polymyxin B, so it excluded the possible contribution of contaminating LPS in the cell stimulation.

These data indicated that SspA-1, as the effector of the T4SS, triggered the excessive secretion of proinflammatory cytokines both *in vivo* and *in vitro*.

### SspA-1-induced immune response and protection of mice against *S. suis* 2

**Figure 5:**
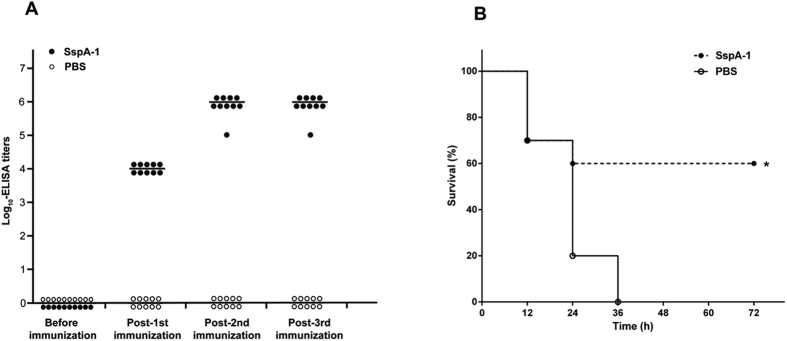
Immune response and survival curves of mice immunized with PBS or recombinant SspA-1. (**A**) SspA-1 specific antibody titers for individual mice immunized with PBS or recombinant SspA-1; the average titer is represented as a bar. (**B**) Survival curves of mice immunized with PBS or recombinant SspA-1 challenged with the wild-type strain 05ZYH33, *P < 0.05.

The mice immunized with SspA-1 elicited a specific humoral IgG response. By contrast, SspA-1-specific antibody titers were below the limit of detection in the sera of the non-immunized mice (Fig. 5A). When the mice were infected with *S. suis* 2, the mice in the non-immunized control group exhibited typical clinical signs and died within 36 h; by contrast, 60% of the SspA-1-immunized mice manifested only mild clinical signs and survived the infection (Fig. 5B). These results indicated that the SspA-1-induced immune response protects the mice against *S. suis* 2 infection.

## Discussion

Although T4SS is implicated in the pathogenicity of the highly virulent *S. suis* 2^13^,^27^, the exact effectors secreted by this system have yet to be identified. In our study, seven candidate T4SS effectors were identified through LC-MS/MS. Among these seven candidates, two subtilisin-like serine proteases (SspA-1 and SspA-2) were selected for further analysis because subtilisin-like serine proteases often act as virulence determinants in certain bacteria.

To verify whether SspA-1 and SspA-2 are secreted by the T4SS of *S. suis* 2, we separately determined the levels of the two proteins in the supernatants and whole cells of the wild-type strain and T4SS-deficient mutant (Δ*virD4*–*89K*) through Western blot. The results revealed that the loss of T4SS function almost completely blocked the secretion of SspA-1 and led to abundant accumulation of SspA-1 in the cells of the mutant strain, indicating that the secretion of SspA-1 depends on the T4SS. This finding is consistent with the LC-MS/MS results. Similar to VirD4 of *A. tumefaciens*, the VirD4–89K of *S. suis* 2 is a key component of T4SS, which acts as a coupling factor that binds T4SS substrates or effectors directly prior to delivery into the VirB transmembrane channel^28^,^29^. In the present study, the interaction between SspA-1 and VirD4-89K was verified by pull-down assays. These tests further confirmed that SspA-1 is the effector of T4SS of the highly virulent *S. suis* 2. However, Western blot results also indicated that the secretion of SspA-2 was unaffected by T4SS (data not shown). Because Western blot is much more sensitive than LC-MS/MS, it could detect SspA-2 in the supernatant of Δ*virD4-89K* which was ignored by LC-MS/MS, thereby leading to the inconsistent results of Western blot and LC-MS/MS on SspA-2. As such, we focused on SspA-1.

Moreover, a trace amount of SspA-1 can still be detected in the culture supernatant of the T4SS-deficient mutant (Δ*virD4*–*89K*) (Fig. 1A), suggesting that SspA-1 secretion is not completely blocked when T4SS is inactivated. Hu *et al.*^23^ demonstrated that SspA is also a surface antigen anchored on the cell wall of *S. suis* 2 (SC19 strain). Thus, a trace amount of proteolytic fragments derived from the anchored SspA-1 may be detected inevitably in the supernatant of the VirD4–89K mutants, although the SspA-1 secretion through T4SS is inhibited. Hence, the SspA-1 of *S. suis* 2 may be secreted in two forms; one of these forms is anchored to the cell wall, and the other is released into the extracellular matrix via T4SS.

To our knowledge, SspA-1 is the first effector identified in Gram-positive bacteria that is transported by T4SS, a system generally thought to be used in conjugation to transfer DNA^30^. Gram-negative bacteria deploy dedicated secretion systems (type I to VI) to overcome the outer membrane barrier in the translocation of proteins. Although Gram-positive bacteria are generally considered lacking in outer membrane, their peptidoglycan layer is much thicker than that of Gram-negative microbes, and they can still block the diffusion of proteins greater than 25–50 kDa across the cell envelope^31^,^32^. It is favored by the evidence that boiling *staphylococci* in hot SDS does not release lipoproteins from the murein sacculus whereas advanced puncturing murein with specific hydrolases extracts such lipoproteins^33^. Therefore, Gram-positive bacteria may also require specialized secretion machinery or transport channels to translocate proteins. For instance, the most studied specialized protein export system in some Gram-positive bacteria (e.g. *Mycobacterium tuberculosis* and *Staphylococcus aureus*) is the type VII secretion system which mediates the secretion of some important virulence factors^34^,^35^. Moreover, in *Streptococcus pyogenes*, a microdomain designated as ExPortal is related to the secretion of precursor proteins across the thick peptidoglycan layer^32^,^36^. In our study, the acquisition of T4SS in 89K PAI during evolution enables the highly virulent *S. suis* 2 to secrete SspA-1 across the thick peptidoglycan barrier into the extracellular milieu.

A majority of patients in the two outbreaks of *S. suis* 2 infection in China developed STSS characterized by a severe systemic inflammatory response^37^. Our previous study suggested that T4SS in 89K PAI of *S. suis* 2 secretes unknown effectors that can trigger an excessive inflammatory response and lead to STSS^13^. Furthermore, a member of the subtilase family which shares 29% identities with SspA-1 can reportedly induce massive secretion of IL-1β, IL-6, TNF-α, CXCL8, and CCL5 in macrophages^25^. Therefore, we wondered whether SspA-1 is responsible for STSS caused by *S. suis* 2. The results from the experimental infection of mice showed that deletion of either SspA-1 or VirD4-89K attenuates the virulence of *S. suis* 2. Additionally, the proinflammatory cytokines released in the sera after infection with Δ*sspA-1* or Δ*virD4-89K* were found at much lower levels than those induced by a wild-type infection. And *in vitro* study also showed that purified SspA-1 could induce the secretion of IL-6, TNF-α, and IL-12p70 in THP-1 cells directly. These data provide further evidence that SspA-1 is an effector of T4SS and enhances the virulence of *S. suis* 2 by triggering an excessive inflammatory response, thereby contributing to the development of STSS. In addition, the protective role of SspA-1-induced immune response suggests that SspA-1 might be a potential vaccine candidate.

The Δ*sspA-1* mutant seems slightly more virulent than the Δ*virD4-89K* mutant on the basis of their survival rates and cytokine stimulation, although the difference is not significant. This finding implies that other T4SS effectors may exist in *S. suis* 2, or T4SS is independently pathogenic, as in the case of *Helicobacter pylori*^38^,^39^. Notably, the reliance on a single culture condition may restrict the discovery of effectors induced in alternative environments or *in vivo*. Considering that T4SS plays a role in effector transport, these possibilities should be further investigated to understand the pathogenic mechanisms of *S. suis* 2.

## Additional Information

**How to cite this article**: Yin, S. *et al.* Subtilisin-like protease-1 secreted through type IV secretion system contributes to high virulence of *Streptococcus suis* 2. *Sci. Rep.* **6**, 27369; doi: 10.1038/srep27369 (2016).

## Supplementary Material

Supplementary Information

## Figures and Tables

**Table 1:** Putative effectors of T4SS by shotgun proteomic analysis.

Locus	GI	Mass	Score	No. of peptides matched (% coverage)	Product	Signal peptide position
05SSU1982	gi|146319636	186968	105	8(6)	Subtilisin-like serine protease (SspA-1)	1–40
05SSU0552	gi|146318206	31367	50	3(2)	Amino acid ABC transporter substrate-binding protein	1–30
05SSU0811	gi|146318465	31665	41	4(3)	Subtilisin-like serine protease (SspA-2)	1–19
05SSU2133	gi|146319787	44302	27	1(1)	Sugar ABC transporter substrate-binding protein	1–34
05SSU1311	gi|146318965	117795	22	19(1)	Hypothetical protein	1–32
05SSU1961	gi|146319615	68516	16	9(1)	Prolyl-tRNA synthetase	None
05SSU0214	gi|146317870	137100	14	2(1)	Sugar ABC transporter periplasmic protein	1–25

## References

[b1] Kudva R. *et al.* Protein translocation across the inner membrane of Gram-negative bacteria: the Sec and Tat dependent protein transport pathways. Res Microbiol 164, 505–534 (2013). 23567322 10.1016/j.resmic.2013.03.016

[b2] Preston G. M., Studholme D. J. & Caldelari I. Profiling the secretomes of plant pathogenic Proteobacteria. FEMS Microbiol Rev 29, 331–360 (2005). 15808747 10.1016/j.femsre.2004.12.004

[b3] Hood R. D. *et al.* A type VI secretion system of Pseudomonas aeruginosa targets a toxin to bacteria. Cell Host Microbe 7, 25–37 (2010). 20114026 10.1016/j.chom.2009.12.007 PMC2831478

[b4] Freudl R. Leaving home ain’t easy: protein export systems in Gram-positive bacteria. Res Microbiol 164, 664–674 (2013). 23541477 10.1016/j.resmic.2013.03.014

[b5] Voth D. E., Broederdorf L. J. & Graham J. G. Bacterial Type IV secretion systems: versatile virulence machines. Future Microbiol 7, 241–257 (2012). 22324993 10.2217/fmb.11.150 PMC3563059

[b6] Trokter M., Felisberto-Rodrigues C., Christie P. J. & Waksman G. Recent advances in the structural and molecular biology of type IV secretion systems. Curr Opin Struct Biol 27, 16–23 (2014). 24709394 10.1016/j.sbi.2014.02.006 PMC4182333

[b7] Gottschalk M., Xu J., Calzas C. & Segura M. *Streptococcus suis*: a new emerging or an old neglected zoonotic pathogen? Future Microbiol 5, 371–391 (2010). 20210549 10.2217/fmb.10.2

[b8] Segura M. *Streptococcus suis*: an emerging human threat. J Infect Dis 199, 4–6 (2009). 19016626 10.1086/594371

[b9] Tang J. *et al.* Streptococcal toxic shock syndrome caused by *Streptococcus suis* serotype 2. PLos Med 3, e151 (2006). 16584289 10.1371/journal.pmed.0030151 PMC1434494

[b10] Ye C. *et al.* *Streptococcus suis* sequence type 7 outbreak, Sichuan, China. Emerg Infect Dis 12, 1203–1208 (2006). 16965698 10.3201/eid1208.060232 PMC3291228

[b11] Chen C. *et al.* A glimpse of streptococcal toxic shock syndrome from comparative genomics of *S. suis* 2 Chinese isolates. PLos One 2, e315 (2007). 17375201 10.1371/journal.pone.0000315 PMC1820848

[b12] Li M. *et al.* GI-type T4SS-mediated horizontal transfer of the 89K pathogenicity island in epidemic *Streptococcus suis* serotype 2. Mol Microbiol 79, 1670–1683 (2011). 21244532 10.1111/j.1365-2958.2011.07553.x PMC3132442

[b13] Zhao Y. *et al.* Role of a type IV-like secretion system of *Streptococcus suis* 2 in the development of streptococcal toxic shock syndrome. J Infect Dis 204, 274–281 (2011). 21673039 10.1093/infdis/jir261

[b14] Wu H. Y., Chung P. C., Shih H. W., Wen S. R. & Lai E. M. Secretome analysis uncovers an Hcp-family protein secreted via a type VI secretion system in *Agrobacterium tumefaciens*. J Bacteriol 190, 2841–2850 (2008). 18263727 10.1128/JB.01775-07 PMC2293243

[b15] Lee C. L. *et al.* Strategic shotgun proteomics approach for efficient construction of an expression map of targeted protein families in hepatoma cell lines. Proteomics 3, 2472–2486 (2003). 14673797 10.1002/pmic.200300586

[b16] Geng H. *et al.* Identification and characterization of novel immunogenic proteins of *Streptococcus suis* serotype 2. J Proteome Res 7, 4132–4142 (2008). 18630869 10.1021/pr800196v

[b17] Li M. *et al.* SalK/SalR, a two-component signal transduction system, is essential for full virulence of highly invasive *Streptococcus suis* serotype 2. PLos One 3, e2080 (2008). 18461172 10.1371/journal.pone.0002080 PMC2358977

[b18] Zhang A. *et al.* Identification of three novel *in vivo*-induced expressed antigens during infection with *Streptococcus suis* serotype 2. FEMS Microbiol Lett 295, 17–22 (2009). 19473247 10.1111/j.1574-6968.2009.01574.x

[b19] Liu F., Wu X., Li L., Liu Z. & Wang Z. Expression, purification and characterization of two truncated peste des petits ruminants virus matrix proteins in *Escherichia coli*, and production of polyclonal antibodies against this protein. Protein Expr Purif 91, 1–9 (2013). 23827209 10.1016/j.pep.2013.06.011

[b20] de Barsy M. *et al.* Identification of a *Brucella* spp. secreted effector specifically interacting with human small GTPase Rab2. Cell Microbiol 13, 1044–1058 (2011). 21501366 10.1111/j.1462-5822.2011.01601.x

[b21] Akeda Y. *et al.* Identification of the *Vibrio parahaemolyticus* type III secretion system 2-associated chaperone VocC for the T3SS2-specific effector VopC. FEMS Microbiol Lett 324, 156–164 (2011). 22092817 10.1111/j.1574-6968.2011.02399.x

[b22] Li Y. Y. *et al.* Immunization with recombinant Sao protein confers protection against *Streptococcus suis* infection. Clinical and Vaccine Immunology 14, 937–943 (2007). 17567767 10.1128/CVI.00046-07 PMC2044494

[b23] Hu Q. *et al.* Identification of a cell wall-associated subtilisin-like serine protease involved in the pathogenesis of *Streptococcus suis* serotype 2. Microb Pathog 48, 103–109 (2010). 19944142 10.1016/j.micpath.2009.11.005

[b24] Bonifait L., Vaillancourt K., Gottschalk M., Frenette M. & Grenier D. Purification and characterization of the subtilisin-like protease of *Streptococcus suis* that contributes to its virulence. Vet Microbiol 148, 333–340 (2011). 21030165 10.1016/j.vetmic.2010.09.024

[b25] Bonifait L. & Grenier D. The SspA subtilisin-like protease of *Streptococcus suis* triggers a pro-inflammatory response in macrophages through a non-proteolytic mechanism. BMC Microbiol 11, 47 (2011). 21362190 10.1186/1471-2180-11-47 PMC3058005

[b26] Bonifait L. *et al.* The cell envelope subtilisin-like proteinase is a virulence determinant for *Streptococcus suis*. BMC Microbiol 10, 42 (2010). 20146817 10.1186/1471-2180-10-42 PMC2832634

[b27] Zhong Q. *et al.* A functional peptidoglycan hydrolase characterized from T4SS in 89K pathogenicity island of epidemic *Streptococcus suis* serotype 2. BMC Microbiol 14, 73 (2014). 24655418 10.1186/1471-2180-14-73 PMC3974602

[b28] Fronzes R., Christie P. J. & Waksman G. The structural biology of type IV secretion systems. Nat Rev Microbiol 7, 703–714 (2009). 19756009 10.1038/nrmicro2218 PMC3869563

[b29] Niu H., Kozjak-Pavlovic V., Rudel T. & Rikihisa Y. *Anaplasma phagocytophilum* Ats-1 Is Imported into Host Cell Mitochondria and Interferes with Apoptosis Induction. PLos Pathogens 6, e1000774 (2010). 20174550 10.1371/journal.ppat.1000774 PMC2824752

[b30] Goessweiner-Mohr N., Arends K., Keller W. & Grohmann E. Conjugative type IV secretion systems in Gram-positive bacteria. Plasmid 70, 289–302 (2013). 24129002 10.1016/j.plasmid.2013.09.005 PMC3913187

[b31] Snyder A. & Marquis H. Restricted translocation across the cell wall regulates secretion of the broad-range phospholipase C of *Listetia monocytogenes*. J Bacteriol 185, 5953–5958 (2003). 14526005 10.1128/JB.185.20.5953-5958.2003 PMC225021

[b32] Rosch J. & Caparon M. A microdomain for protein secretion in Gram-positive bacteria. Science 304, 1513–1515 (2004). 15178803 10.1126/science.1097404

[b33] Navarre W. W., Daefler S. & Schneewind O. Cell wall sorting of lipoproteins in *Staphylococcus aureus*. J Bacteriol 178, 441–446 (1996). 8550464 10.1128/jb.178.2.441-446.1996 PMC177676

[b34] Abdallah A. M. *et al.* Type VII secretion - mycobacteria show the way. Nature Reviews Microbiology 5, 883–891 (2007). 10.1038/nrmicro1773 17922044

[b35] Burts M. L., Williams W. A., DeBord K. & Missiakas D. M. EsxA and EsxB are secreted by an ESAT-6-like system that is required for the pathogenesis of *Staphylococcus aureus* infections. Proc Natl Acad Sci USA 102, 1169–1174 (2005). 15657139 10.1073/pnas.0405620102 PMC545836

[b36] Rosch J. W. & Caparon M. G. The ExPortal: an organelle dedicated to the biogenesis of secreted proteins in *Streptococcus pyogenes*. Mol Microbiol 58, 959–968 (2005). 16262783 10.1111/j.1365-2958.2005.04887.x

[b37] Ye C. *et al.* Clinical, experimental, and genomic differences between intermediately pathogenic, highly pathogenic, and epidemic *Streptococcus suis*. J Infect Dis 199, 97–107 (2009). 19016627 10.1086/594370

[b38] Tegtmeyer N., Wessler S. & Backert S. Role of the cag-pathogenicity island encoded type IV secretion system in *Helicobacter pylori* pathogenesis. FEBS J 278, 1190–1202 (2011). 21352489 10.1111/j.1742-4658.2011.08035.x PMC3070773

[b39] Koch M., Mollenkopf H. J., Klemm U. & Meyer T. F. Induction of microRNA-155 is TLR- and type IV secretion system-dependent in macrophages and inhibits DNA-damage induced apoptosis. Proc Natl Acad Sci USA 109, E1153–1162 (2012). 22509021 10.1073/pnas.1116125109 PMC3358876

