# Supplementary material for: Subtilisin-like protease-1 secreted through type IV secretion system contributes to high virulence of Streptococcus suis 2
Source: Sci Rep. 2016 Jun 8;6:27369. doi: 10.1038/srep27369 (PMC4897608; doi:10.1038/srep27369)
Supplement: Supplementary Information [file srep27369-s1.pdf]

Subtilisin-like protease-1 secreted through type IV secretion system contributes to high virulence of *Streptococcus suis* 2

Supeng Yin[1], Ming Li[1], Xiancai Rao[1], Xinyue Yao[1], Qiu Zhong[1], Min Wang[1], Jing Wang[1], Yizhi Peng[2], Jiaqi Tang[3], Fuquan Hu[1*], Yan Zhao[1*]

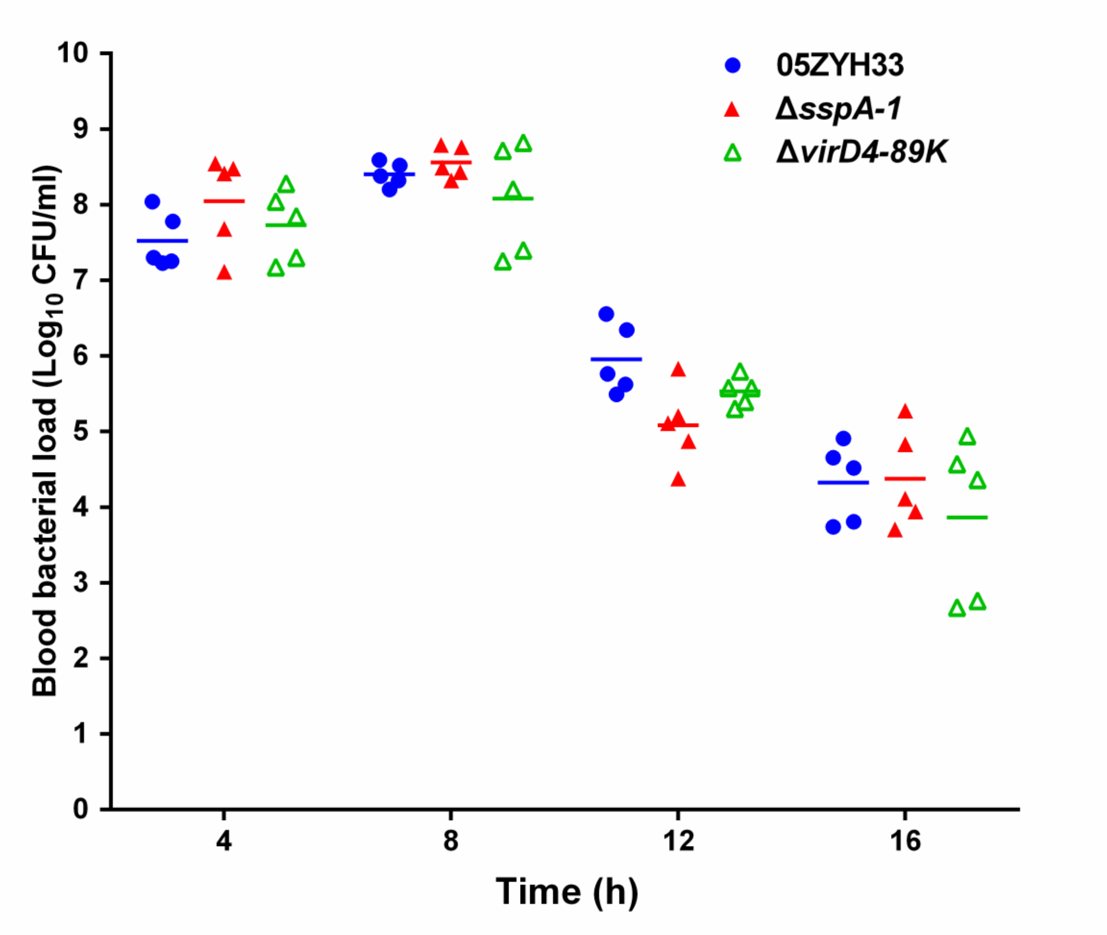

Figure S1. Blood bacterial loads of infected mice.

The blood bacterial loads in the BALB/c mice infected with the designated strains at 4, 8, 12, and 16 h after infection. $P > 0.05$ for the comparison among the wild-type strain 05ZYH33, Δ*sspA-1* mutant, and Δ*virD4–89K* mutant at each of the same time point. The data are representative of three independent experiments.

Table S1. Bacterial strains and plasmids

Strains/plasmids	Characteristics/Function	Source
**Strains**		
05ZYH33	A highly virulent strain isolated from a deceased patient with a streptococcal toxic shock syndrome	Lab collection
Δ*sspA-1*	05ZYH33 derivative with the *sspA-1* gene replaced by a *spc* gene cassette	This study
CΔ*sspA-1*	Complemented strain of Δ*sspA-1*; Spc^R; Em^R	This study
Δ*virD4-89K*	05ZYH33 derivative with the *virD4-89K* gene replaced by a *spc* gene cassette	Lab collection
Δ*virB1-89K*	05ZYH33 derivative with the *virB1-89K* gene replaced by a *spc* gene cassette	Lab collection
Δ*virB4-89K*	05ZYH33 derivative with the *virB4-89K* gene replaced by a *spc* gene cassette	Lab collection
E. coli DH5α	Cloning host for maintaining recombinant plasmids	Lab collection
E. coli BL21(DE3)	Expression host for overproducing recombinant protein	Lab collection
**Plasmids**		
pET-28a	His-tag fusion expression vector ; Kan^R	Novagen
pGEX-6P-1	GST-tag fusion expression vector; Amp^R	GE Healthcare
pUC18	Cloning vector; Amp^R	TaKaRa
pVA838	*E. coli*-*S. suis* shuttle vector; Em^R; Cm^R	Lab collection

Spc^R, spectinomycin resistant; Kan^R, kanamycin resistant; Amp^R, ampicillin resistant; Em^R, erythromycin resistant.

Table S2. Primers used in this study

Primers	Sequence (5’-3’)	Function
LA-F	CCGGAATTCAGTGGTCTCAGATTGAG (*Eco*R I)	*sspA-1* knock out
LA-R	CGCGGATCCCGTGGGTATCATCGTAGAGA (*Bam*H I)	*sspA-1* knock out
RA-F	AACTGCAGAAGTTTGGGAGGTTCGAG (*Pst* I)	*sspA-1* knock out
RA-R	CCCAAGCTTGAATGAGCTTGTTCG (*Hin*d III)	*sspA-1* knock out
spc-F	CGCGGATCCGTTCGTGAATACATGTTATAATA (*Bam*H I)	*sspA-1* knock out
spc-R	AACTGCAGTTTCTAAAATCTGAT (*Pst* I)	*sspA-1* knock out
In-F	TCAATCGCCTCTCAATCCAG	*sspA-1* knock out
In-R	CCTCCCAAACTTTATTATTATCC	*sspA-1* knock out
Out-F	AGCCTGTGCTTTTTCTTGTT	*sspA-1* knock out
Out-R	TTCACTATCCACTACCTGTCTT	*sspA-1* knock out
C*sspA-1*-F	CGCGGATCCGTTGGCGACACTGTTTGTATTATT (*Bam*H I)	*sspA-1* complement
C*sspA-1*-MR	ACATGCATGCCCAGATGAATCTAGAAC (*Sph* I)	*sspA-1* complement
C*sspA-1*-MF	ACATGCATGCTTTGGAAAATTCTCC (*Sph* I)	*sspA-1* complement
C*sspA-1*-R	CCGGAATTCTTACATTGGTATATGCGCTTCCGAT (*Eco*R I)	*sspA-1* complement
sspA-in-F	TCAATCGCCTCTCAATCCAG	qRT-PCR
sspA-in-R	CCTCCCAAACTTTATTATTATCC	qRT-PCR
16S-in-F	GTTGCGAACGGGTGAGTAA	qRT-PCR
16S-in-R	TCTCAGGTCGGCTATGTATCG	qRT-PCR
sspA-F	GGAATTCCATATGGGAGTGGTCGAAGAAGTTGT (*Nde* I)	His-SspA-1 expression
sspA-R	CCCTCGAGAGAACTGGTTTCCCAAGCC (*Xho* I）	His-SspA-1 expression
virD4-F	CGCGGATCCACAGGACAGAAGGTCTATCG (*Bam*H I）	GST-VirD4 expression
virD4-R	CCGCTCGAGTTAATGTAGTGTCGTTTCTGTGC (*Xho* I)	GST-VirD4 expression
